# The Attitude of Great Writers Towards Disease

**Published:** 1893-08-26

**Authors:** 


### The Attitude of Great Writers Towards Disease.

### PEPYS ON THE GREAT PLAGUE.

We possess several accounts of great plagues written by persons who were eye-witnesses of what they relate. They give very vivid pictures of all that went on from a plague point of view, and describe very forcibly the effect upon the people in general of the near dread of death. But they generally give a very imperfect picture of everyday life under these terrible circumstances; for even in plague time the business of the town did not consist wholly of sickness and burying. And they write as a rule objectively. The emotions are those of their neighbours depicted from a standpoint of passive observation.

Now Pepys gives no very detailed account of the Plague of 1665. Of actual description there is scarcely a trace in his Diary. But his talk about it is unique in this, that it gives a faithful picture of the impression which it all made upon him individually. The very want of coherent plan in the Diary is an earnest of its holding up a true mirror to its writer's thoughts. In the midst of grave fears that the kingdom be wholly lost, will come skimming to the surface the memory of the "little rent in my fine new camlet cloak," darned up at the tailor's to show no blemish. All the prevailing interests of the day we are sure of finding noted. Nothing is kept back from a desire to be dignified; nor is he ever afraid of looking his thoughts in the face. And the story of what is uppermost in his mind, always curious, is specially instructive and characteristic through these months of terror.

The first mention of the Plague in the Diary is on April 30th, 1665, where his little summary of the month's events thus ends: "Great fears of the sicknesse here in the City, it being said that two or three houses are already shut up. God preserve us all!" For more than three weeks the subject dies out. He is full of a visit to Greenwich and the "tryal of some experiments about making of coaches easy." At Mr. Evelyn's, too, he is much taken with a hive of bees in glass, "so as you may see the bees making honey and combs mighty pleasantly." On Whit-Sunday he is at leisure to admire his wife, "very fine in a new yellow bird's-eye hood." And then he becomes absorbed in the affairs of the fleet, till on May 24th we read that the talk of the coffee-house is "of the Plague growing upon us in this town; and of remedies against it, some saying one thing and some another." It has not yet been brought near to him, and for another fortnight the little events of the then London season absorb him entirely. A scandalous story of my Lord Rochester and a grand funeral at which he appears in "my new camelot suit, the best that ever I wore in my life, costing me above £24," are the chief events of the interval. But on June 7th he receives a shock. "This day, much against my will, I did in Drury Lane see two or three houses marked with a red cross upon the doors, and 'Lord have mercy upon us' writ there; which was a sad sight to me, being the first of the kind that to my remembrance I ever saw." A few days after it is brought home to him even more emphatically by seeing the house of his friend and physician, Dr. Burnett, shut up in a similar manner; "he discovered it himself first, and caused himself to be shut up of his own accord, which was very handsome." And now in the middle of June "the town grows very sickly and people to be afraid of it," and the record of weekly deaths from the Plague begins, only 112 so far, but that is more than double the preceding return. At Court all goes on as usual. Pepys visits the Duke and his courtiers at Whitehall, returned from sea. "All fat and lusty and ruddy by being in the sun." The Dutch war and the doings of the fleet are still the prevailing topics. Later on in the month "I find all the town almost going out of town, the coaches and waggons being all full of people going into the country." Yet it is confidently believed that the Plague is already decreasing.

And now we come to a topic which is to afford Mr. Pepys his greatest diversion of thought in an anxious time. Already in February there has been some hint of a projected marriage between "my Lady Jemimah" (Lord Sandwich's daughter) and Sir G. Carteret's eldest son. The matter had smouldered from some doubt as to the amount of landed property possessed by the gentleman. By the end of June that doubt seems to have been satisfactorily settled, and Pepys is commissioned by my Lord Sandwich to open the matter to the proposed bridegroom. From this time the Plague and the love affair mingle oddly in the Diary. Sometimes one is uppermost, sometimes the other. Both are comprehended in the little review of his circumstances which ends the month. "Thus this book of two years ends. Myself and family in good health. . . In a sickly time of the Plague growing on. All other things well, especially a new interest I am making by a match in hand between the eldest son of Sir G. Carteret and my Lady Jemimah Montagu." The Plague grows steadily worse through July. The park is shut up, nearly all the fashions being out of town. The weekly deaths increased from 700 to 1,700, and a solemn fast for the Plague is observed. A curious fact bearing upon the sanitary condition of the City at that time is noted that "in this parish of Michell's Cornhill there hath, notwithstanding this sickliness, been buried of any disease, man, woman, or child, not one for thirteen months last past." Through it all the little romance with its oddly businesslike accompaniments continues, and preparations for "my Lady Jem.'s wedding" are in progress before Mr. Carteret has paid his first visit to the proposed bride. When the important visit is made Pepys accompanies the young man. "But Lord! what silly discourse we had as to love matters, he being the most awkerd man ever I met with in my life as to that business." Pepys advices against his being left alone with the lady having evidently some misgivings she may find him too dull. Mr. Carteret professes after the first evening spent in her society that he likes the lady mightily: "but, Lord! in the dullest insipid manner that ever lover did." The next morning, the Lord's Day, they are to appear at church together, and Mr. Carteret is instructed by his faithful mentor "what to do: to take the lady always by the hand to lead her, and that he should make these and these compliments." It is disheartening after this to find Mr. Carteret returning from the "pretty good sermon," without having had the confidence to take his lady once by the hand, coming or going, "which I told him of when we come home, and he will hereafter do it." A heavy young man to be sure. It is not surprising that the young lady was not very enthusiastic about the match, but she brings herself to say "she could readily obey what her father and mother had done," and so all goes smoothly. Pepys' interest in the affair appears to have been that of the genuine busybody, or, perhaps, the feeling which he describes at another wedding, "strange to see what delight we have to see these poor fools decoyed into our condition"; the only present he gets for his trouble is a bottle of plague water from my Lady Carteret. He is not unmindful as a prudent man of his danger in the surrounding sickness, and learning that it is got into his parish, begins to think of setting things in order, "which I pray God enable me to put both as to soul and body." By the end of the month the young couple are now well acquainted. Lord Sandwich's chaplain is dead of the plague, and Mr. Pepys finds himself an unwelcome guest at the house on account of the infection he may bring from the City. "These considerations make us all hasten the marriage." And accomplished it is on July 31st, "I being in my new coloured silk suit"; "they being both in their old clothes." "And very merry we were, but in such a sober way as never almost anything was in so great families." Cause enough there was to be sober, yet the Diary closes the month with the record, "Thus I ended this month with the greatest joy that ever I did any in my life. . . . After the greatest glut of content that ever I had, only under some difficulty because of the Plague."

It was not yet at its height. For some weeks the number of deaths rose till the climax appears to have been reached about the middle of September. Pepys remained in town himself till the end of August, and then joined his wife at Woolwich for a month or two. He continues throughout to do business as usual at his office, to visit his friends, buy new clothes, and amuse himself with gossip about the Court and his neighbours. It is astonishing always how small a proportion of his attention is absorbed by the prevailing infection, and it is impossible not to see also that in this he was in no way different from other people. Through the winter the Plague lingered, now more, now less, but people gradually returned to town; shops were opened cautiously, and the streets though still empty showed signs of life. Pepys, who had never shown the slightest symptom of panic, is maliciously amused at the panic of some who should have known better. At the first meeting of Gresham College in January he notes: "Dr. Goddard did fill us with talk in defence of his and his fellow physicians going out of town in the Plague-time," and on February 4th we hear of "the very poor and bad excuse" made by the parson, Mr. Mills, "for his leaving the parish before anybody went, and now staying till all are come home."

On the whole, few persons cut a better figure through this dark time than Pepys himself, who with the knowledge ever present with him that a man could not reckon upon living two days, stuck steadily to his duties, and found in obliging his friends his principal diversions.

